# Quantifying signaling pathway activation to monitor the quality of induced pluripotent stem cells

**DOI:** 10.18632/oncotarget.4673

**Published:** 2015-08-22

**Authors:** Eugene Makarev, Kristen Fortney, Maria Litovchenko, Karl H. Braunewell, Alex Zhavoronkov, Anthony Atala

**Affiliations:** ^1^ Atlas Regeneration, Inc, Winston-Salem, NC, USA; ^2^ Insilico Medicine, Inc, ETC, Johns Hopkins University, Baltimore, MD, USA; ^3^ Department of Developmental Biology, Stanford University Medical Center, Stanford, CA, USA; ^4^ Department of Computational Genomics, Ludwig Maximilian University of Munich, Germany; ^5^ Department of Neurophysiology, Medical Faculty, Ruhr University Bochum, Germany; ^6^ The Biogerontology Research Foundation, London, UK; ^7^ Department of Urology, Wake Forest Institute for Regenerative Medicine, Wake Forest School of Medicine, Winston-Salem, NC, USA

**Keywords:** Gerotarget, bioinformatics, algorithm, embryonic stem cells, pathway activation, iPSC

## Abstract

Many attempts have been made to evaluate the safety and potency of human induced pluripotent stem cells (iPSCs) for clinical applications using transcriptome data, but results so far have been ambiguous or even contradictory. Here, we characterized stem cells at the pathway level, rather than at the gene level as has been the focus of previous work. We meta-analyzed publically-available gene expression data sets and evaluated signaling and metabolic pathway activation profiles for 20 human embryonic stem cell (ESC) lines, 12 human iPSC lines, five embryonic body lines, and six fibroblast cell lines. We demonstrated the close resemblance of iPSCs with ESCs at the pathway level, and provided examples of how pathway activity can be applied to identify iPSC line abnormalities or to predict *in vitro* differentiation potential. Our results indicate that pathway activation profiling is a promising strategy for evaluating the safety and potency of iPSC lines in translational medicine applications.

## INTRODUCTION

The discovery of human induced pluripotent stem cells (iPSCs) in 2006 completely revolutionized the field of stem cell biology, and these cells are expected to play a key role in the development of many regenerative medicine therapies. IPSC lines can be obtained by reprogramming somatic cells using ectopic expression of transcription factors, such as OCT4, SOX2, KLF4, and C-MYC or alternative reprogramming cocktails [1, 2]. Similar to ESCs they can be cultured and expanded for many passages *in vitro* and are able to differentiate into all three embryonic germ layers. IPSC lines are powerful tools which can be used to study human embryonic development [3], as model systems for human diseases, and as a renewable source for regenerative medicine [4]. Promising applications for human disease modeling include generation of iPSCs derived from methyl CpG binding protein 2 (MeCP2)-deficient patients with RETT syndrome, an autism spectrum disorder, and from a single Parkinson's disease patient harboring a mutation in the leucine-rich repeat kinase 2 gene (LRRK2) [5, 6]. The first human clinical trial of iPSCs was started by Takahashi and colleagues at the RIKEN Center for Developmental Biology in Kobe in 2013 for treating age-related macular degeneration using human iPSC-derived retinal pigment epithelium cell sheets [6, 7].

These applications require the selection and characterization of iPSC lines that reliably, efficiently, and stably differentiate into disease-relevant cell types. However, despite the fact that iPSCs theoretically have the full potential of embryonic stem (ES) cells, there are several studies showing evidence that induction of iPSCs can generate cell lines with genetic or epigenetic variability and abnormalities that could affect iPSC differentiation potential and clinical safety [9–15]. Lister et al. [14] demonstrated significant epigenetic reprogramming variability, such as aberrant reprogramming of DNA methylation, in iPSCs. Hu et al. [15] showed that human iPSCs generate neural epithelium at a significantly reduced efficiency and increased variability compared to hESCs, and suggested that additional differentiation assays will enable to select more uniform iPSC lines [15].

These findings raised the question of the variability of iPSC lines and whether iPSCs and ESCs are fully equivalent. To address these concerns, many recent attempts have been made to evaluate the safety and potency of iPSCs using genome-wide readouts of cell function, such as transcriptomes and methylomes, however, the results are often ambiguous and sometimes contradictory [16, 17]. For instance, Mallon et al. [16] found no significant differences between several isogenic hiPSCs lines derived from the (WA01) hESC line and the original ESC line at the gene expression and methylation level. In contrast, Bock et al. [17] demonstrated that epigenetic and transcriptional variations are common among ESCs and iPSCs and that this variation can have significant impact on the usability of iPSC lines. Thus, potential differences between individual ESC and iPSC lines must be better controlled before these lines are used for translational research and regenerative medicine.

To address the issue of variability and safety in iPSCs, Bock et al. previously established a reference set of DNA methylation maps and gene expression profiles for 20 human ESC lines, 12 human iPSC lines, five embryonic body lines, and six fibroblast cell lines, and further provided data on the *in vitro* differentiation capacity of these ESC and iPSC lines [17]. While this and other previous studies have used transcriptome data to characterize stem cells at the level of individual genes, we used these data (available from GSE25970) to apply a new bioinformatics approach, Regeneration Intelligence, to address the quality issues at the pathway level. We implemented pathway activation scoring (PAS) algorithms [18], which can quantitatively measure the activation of intracellular signaling pathways based on transcriptomic data in cells and tissues in different physiological or pathological conditions, including cancer [19, 20]. Pathway activation scoring was first successfully used to analyze gene expression datasets for nine different human cancer types [19]. In that study, PAS values for 82 signaling pathways were shown to yield superior cancer-specific biomarkers, with better area under the curve (AUC) values and higher predictive power compared to individual genes. Pathway activation scoring analysis has also been applied to study ageing and age-related macular degeneration [21, 22], and to predict cetuximab sensitivity in human colorectal cancers [23]. Using a set of bioinformatics algorithms and tools, termed OncoFinder, allowed the characterization of the functional states of transcriptomes and interactomes accurately, and has the advantage to significantly reduce the errors introduced by transcriptome-wide experimental techniques [24].

Here, we defined the typical pathway profiles of iPSCs *vs*. ESCs from the GSE25970 data set and the resemblance of iPSC with ESC lines at the PAS level in comparison with EB and fibroblast lines. Additionally, we provided examples of how iPSC line abnormalities can be identified using our bioinformatics algorithm, and how pathway activation correlated with the known iPSC differentiation potential. We showed that the PAS algorithm can provide a powerful bioinformatics tool for quality control of iPSC lines prior to clinical application.

## RESULTS

### Pathway Activation Score (PAS) distribution in ESC and iPSC lines

**Figure 1:**
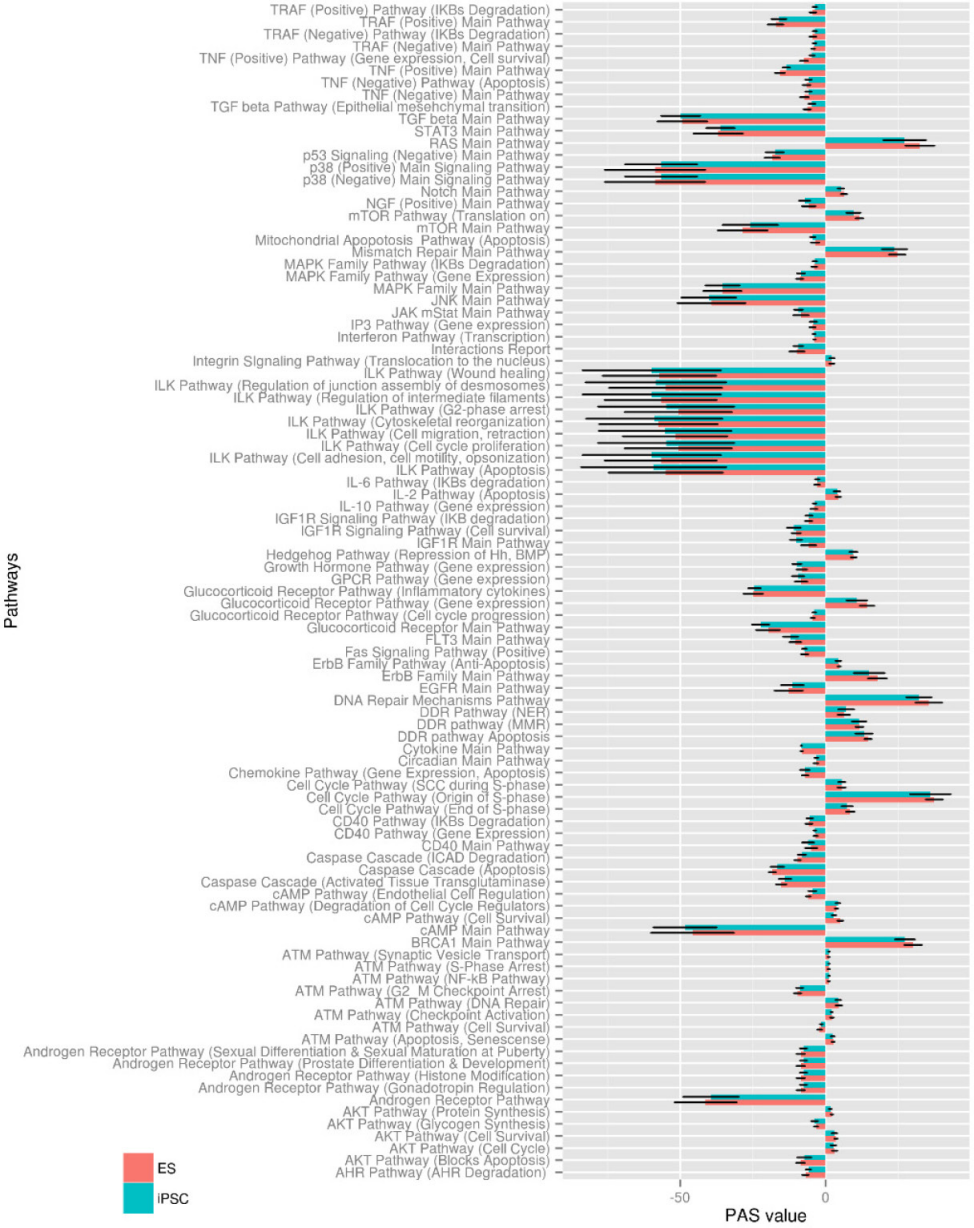
Distribution of statistically significant PAS in ESC and iPSC lines PAS values were calculated with the Regeneration Intelligence software suite for ESC (orange) and iPSC (green) cell lines from GSE25970. Mean PAS values ± SD for 97 pathways with *p*-value < 0.001 are shown.

NCBI Gene Expression Omnibus (GEO) (http://www.ncbi.nlm.nih.gov/geo/) and EBI ArrayExpress (http://www.ebi.ac.uk/arrayexpress/) databases were searched for the most representative data set to include 10 or more microarray profiled ESC and iPSC lines and any iPSC differentiation assay linked to the profiled iPSC lines. One dataset, which met these requirements, was GSE25970 (http://www.ncbi.nlm.nih.gov/geo/query/acc.cgi?acc=GSE25970) which consists of 20 human ESC lines, 12 human iPSC lines, six fibroblast cell lines, and five embryoid body (EB) cell lines, and includes *in vitro* differentiation data for each iPSC line. Next, we used these data to calculate PAS scores for 271 pathways, including more than 3500 genes, in iPSC and ESC lines. The raw PAS data, including p- and *q*-values, for all 271 pathways in 20 ESC and 12 iPSC lines derived from the GSE25970 dataset are presented (Supplemental Excel data file 1). In all our post-analyses, we restricted our attention to significantly dysregulated pathways with false discovery rate *q* < 0.05, which narrow our results to 215 dysregulated pathways in ESC lines and 205 dysregulated pathways in iPSC lines. The individual PAS distribution values with false discovery rate q < 0.05 for all ESC and iPSC lines are shown in Supplemental Figure 1A and 1B, respectively. To restrict attention to the most significant findings we applied an additional criterion of p < 0.001 and found that from a total of 271 pathways 97 distinct pathways were significantly dysregulated simultaneously in ESC and iPSC lines compared to fibroblast control. The corresponding PAS values (*p* < 0.001) for ESC and iPSC lines shown on Figure 1 as determined using the Regeneration Intelligence software suite. Thus, iPSC and ESC lines displayed very similar PAS profiles with a highly significant value overlap in all 97 pathways.

### PAS variation in ESC and iPSC lines

**Figure 2:**
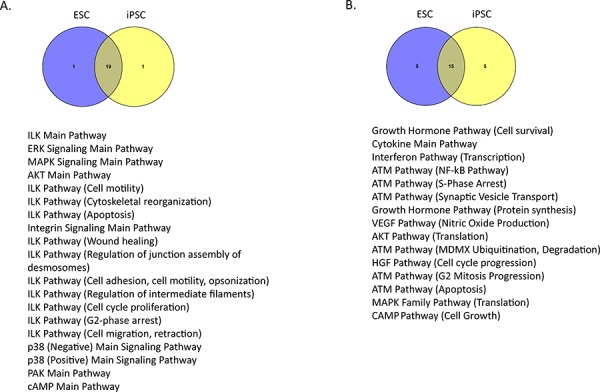
Pathways showing high and low variability within ESC or iPSC lines **A.** The top 20 most variable pathways in ESC and iPSC lines. **B.** The top 20 least variable pathways in iPSC and ESC lines. The most and least variable pathways common to both ESC and iPSC lines are listed.

We identified the top 20 most and least variable pathways in ESC and in iPSC lines (Figure 2). We found that many of these pathways, including the mitogen-activated protein kinase (MAPK), protein kinase B (Akt/PKB), and cyclic adenosine monophosphate (cAMP) signaling pathways, were shared in common between iPSC and ESC lines. In the top 20 most variable pathways, iPSCs and ESC lines had 19 pathways in common (Figure 2A), and in the top 20 least variable pathways the lines shared 15 pathways (Figure 2B). These findings confirm the significant similarity of PAS profiles between groups of iPSCs and ESCs.

### ESC and iPSC lines show similar patterns of pathway activation

**Figure 3:**
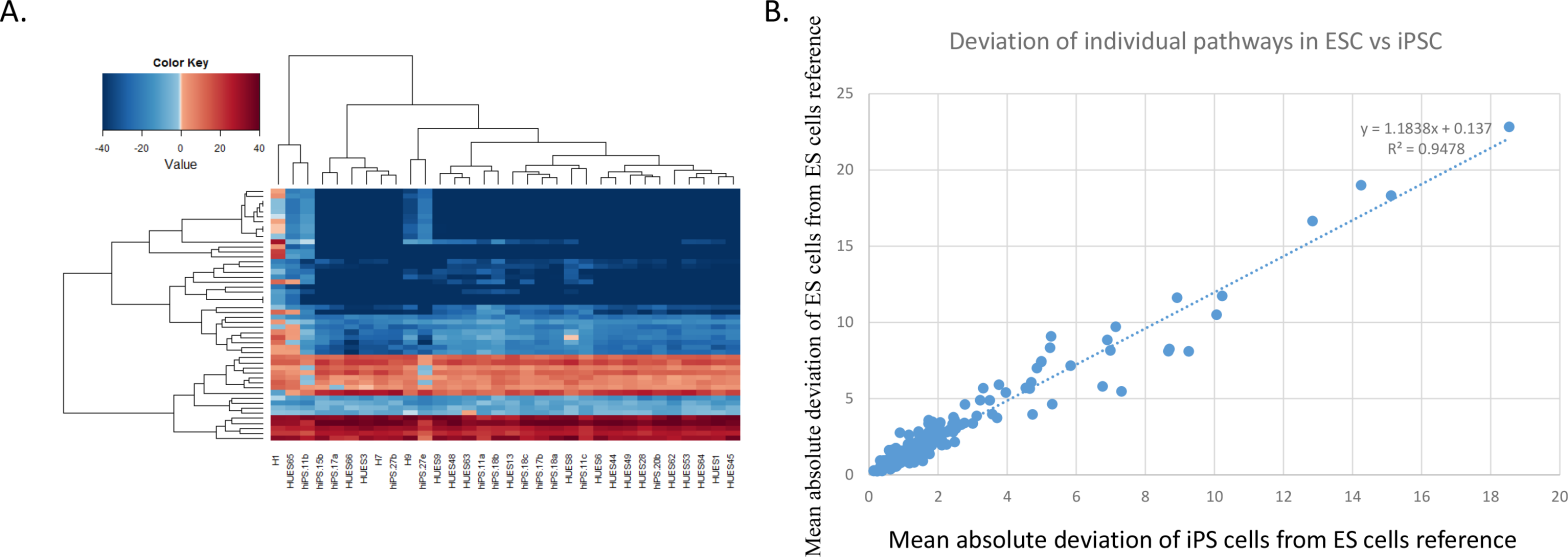
ESC and iPSC lines exhibit a common profile of pathway activation **A.** Hierarchically clustered heat map of the top-50 most variable pathways for all iPSC and ESC lines (using fibroblast cell lines as reference). Blue indicates down-regulation, and red indicates up-regulation. **B.** Scatterplot comparing the deviation of each pathway in ESC *vs*. iPSC lines (measured relative to the ESC references; to calculate deviations for ESC, each ESC line was excluded one at a time from the averaged ES PAS reference to prevent comparing cell lines to themselves).

Next, we clustered the PAS values for all ESC and iPSC lines for the top 50 most variable pathways. This analysis revealed that ES and iPSC lines share a broadly similar pattern of PAS, i.e. ES and iPSC lines do not form separate clusters (Figure 3A). Furthermore, pathways showing the most PAS variability within ES lines also tended to show the most variability within iPSC lines. We calculated the correlation between ESC and iPSC PAS deviation to be R^2^ = 0.9478 (Figure 3B). These results further indicated that the tested iPSCs are very similar to the ESCs at the pathway level.

### A PAS quality score can identify impaired iPSC lines

**Figure 4:**
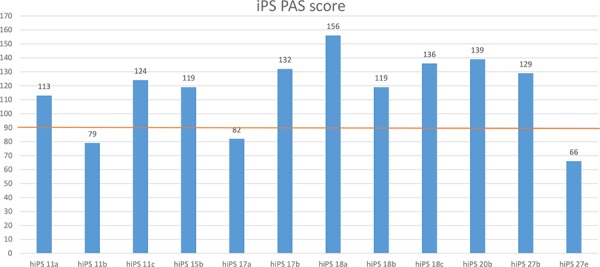
PAS-based quality scores for 12 iPSC lines Shown for each iPSC line is the number of PAS that fall within the ES PAS quality score that are indistinguishable from ES PAS. The upper and lower limits for each ES PAS quality score were calculated as the average of the PAS values across all ESC lines for each pathway, plus or minus SD. The red line corresponds to 50% (95 pathways falling within corresponding ESC PAS quality range) of the maximum (190) iPSC quality score calculated using 190 pathways. Note that, the iPSC lines hiPS11b, hiPS17a, and hiPS27e lines received the lowest PAS-based quality scores; this is consistent with the observation in Bock et al. [17] that hiPS11b and hiPS27e show impaired, whereas hiPS17a showed enhanced differentiation capacity.

In order to develop a quality control tool to identify outlier iPSCs, we defined a “healthy range” of PAS scores built from ESC PAS for each pathway. Specifically, for each pathway we set the upper and lower limit of the ESC PAS quality range to be the average of PAS values ± SD across all ESC lines for each pathway. For each of the twelve iPSC lines we calculated a PAS quality score, defined as the number of PAS values falling within corresponding ESC PAS quality range (Figure 4). This analysis revealed that some iPSC lines have lower quality scores, which may indicate that they have impaired or increased differentiation capabilities. For instance, three lines, hiPS11b, hiPS17a, and hiPS27e displayed lower than average PAS quality scores. The quality score values of these lines were below 50% of the maximum iPSC quality score as calculated using 190 pathways (Figure 4). Consistent with our findings, hiPS27e lacked, hiPS11b showed reduced, whereas hiPS17a showed increased differentiation capacity as compared to ES cells [17]. Since the increased differentiation capacities of hiPS17a cell line could be related to additional pathway dysregulation in hiPS17a cell line that in turn decreases hiPS17a PAS quality score below average. Thus, an iPSC quality score calculated from PAS values derived from transcriptional profiling data can provide a powerful tool for simple and effective iPSC line validation.

### A pathway activation signature that discriminates between iPSCs and fibroblasts

**Figure 5:**
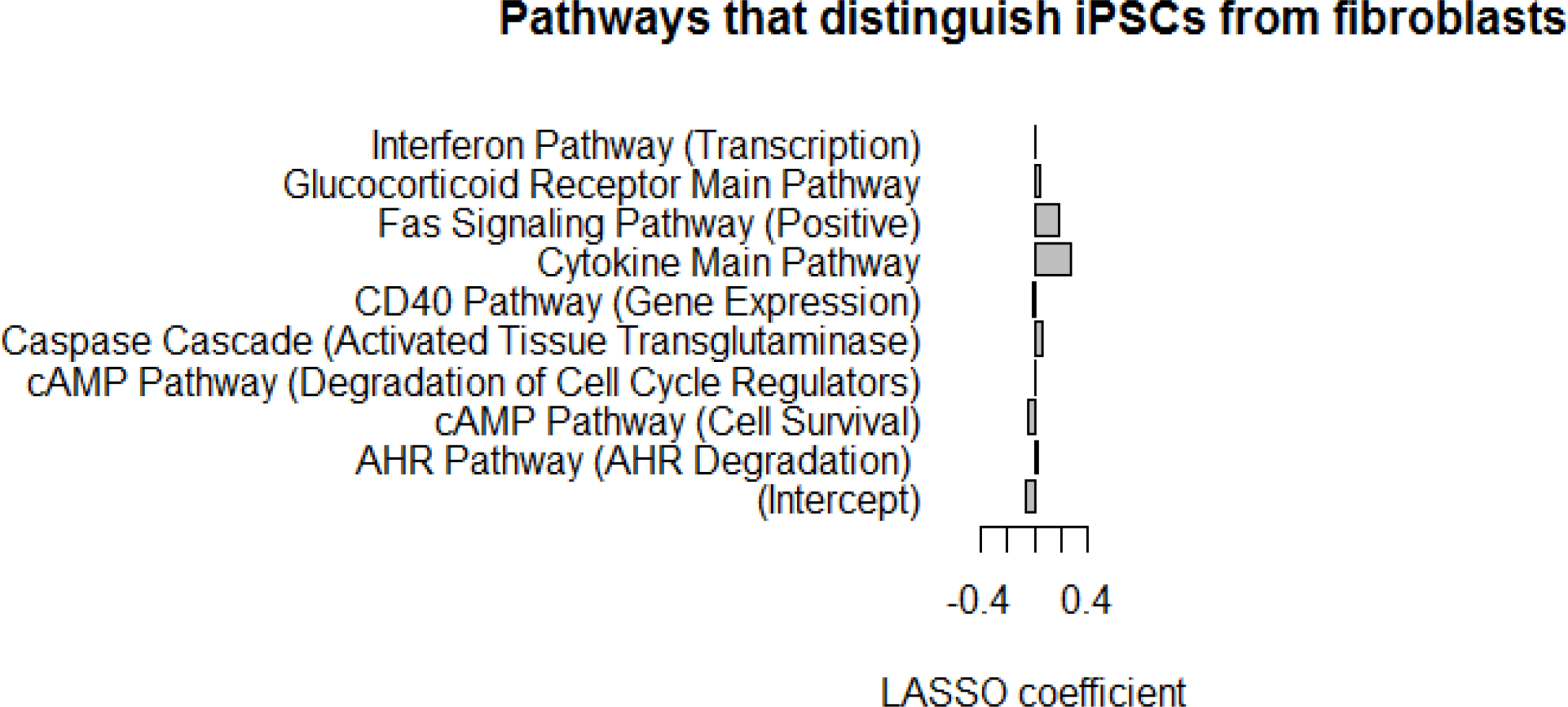
LASSO model using PAS scores can discriminate between iPSCs and fibroblasts Lasso model coefficients for the nine pathways selected by LASSO as strong predictors of iPSC lines *vs*. fibroblasts. Pathways with negative coefficients have lower PAS scores in fibroblasts, and pathways with positive coefficients have higher PAS scores in fibroblasts. Classifier accuracy estimated with cross-validation is 100%.

Finally, we applied the Least Absolute Shrinkage and Selection Operator (LASSO) algorithm [25, 26], which can be used to select predictors of a target variable from a larger set of candidate predictors, to identify individual PAS pathways able to distinguish between iPSCs and fibroblasts, using data from 18 cell lines (12 iPSC and 6 fibroblast cell lines). To estimate model accuracy, we used a leave-one-out cross-validation design where each sample was held out one at a time. For each held out sample, the LASSO model was trained on the 17 remaining samples, and then the trained model was applied to predict the class label of the held out sample. The accuracy over all samples was 100%, indicating that there were clear and strong differences at the pathway level between iPSCs and fibroblasts that are apparent even from a small number of samples. The optimal LASSO model trained on all samples selected nine pathways that together discriminate iPSCs from fibroblasts. The model coefficients for the nine pathways which were selected by LASSO are strong predictors of iPSCs characteristics versus fibroblast characteristics (Figure 5). Pathways with negative coefficients, such as the cAMP pathway (cell survival), had lower PAS scores in fibroblasts, and pathways with positive coefficients, such as the cAMP pathway (degradation of cell cycle regulators), the FAS signaling pathway, and the cytokine main pathway, had higher PAS scores in fibroblasts. In summary, the LASSO model identified a small number of pathways that can reliably distinguish iPSC lines from fibroblasts by examining the PAS values.

## DISCUSSION

We analyzed existing stem cell transcriptome data (GSE25970) to compare iPSCs, ESCs, and fibroblasts at the pathway level. We found that iPSCs and ESCs show a similar pattern of pathway activity that is distinct from the pathway activity of fibroblast and EB cell lines. We defined a new quality score for iPSCs based on PAS, and demonstrated how it can be applied to identify iPSC abnormalities using *in vitro* data. Our results demonstrate that pathway activation, as measured with Regeneration Intelligence, is a promising strategy for iPSC quality control.

Hu et al. [15] demonstrated in human iPSCs in comparison with hESCs that although iPSCs use the same transcriptional network to generate neural epithelium and functionally appropriate neuronal types over the same developmental time course in response to the same set of morphogens, a significantly reduced efficiency and increased variability occurred compared to as hESCs. These results were consistent across various iPSC lines and independent of the use and the set of reprogramming transgenes used to derive iPSCs. Moreover, neural differentiation efficiency of several lines was improved after using additional neural inducers, suggesting that the observed variability in differentiation efficiency depended on different mechanisms, and therefore iPSC lines likely show a distinct variability in their differentiation capability. The authors concluded that in addition to the standard pluripotency assays additional assays, such as differentiation assays to target cell types, may help to select more uniform iPSC lines [15]. Our search criteria for a selecting a useful data set for the development of a bioinformatics algorithm usable for iPSC quality control based on transcriptional data, was to include 10 or more microarray profiled ESC and iPSC lines and iPSC differentiation data linked to the profiled iPSC lines. Out of 101 initial datasets comprising ESC and iPSC data, only the GSE25970 dataset [17] fitted these criteria, and we chose not to combine different available data sets because cross platform normalization methods are not sufficiently reliable for the purpose of iPSC line quality control. In this data set genomic assays and *in vitro* differentiation data were combined into a valuable data set consisting of genome-wide reference maps of gene expression and DNA methylation in a collection of pluripotent cell lines, including 20 human ESC lines, 12 human iPSC lines, six fibroblast, and five EB cell lines, which can be used to analyze the transcriptional properties of these ESC and iPSC lines in comparison with founder lines. By using the genome-wide reference maps of gene expression and DNA methylation in ESC, iPSC, EB, and fibroblast of the GSE25970 data set combined with our algorithm, we were able to demonstrate close resemblance of iPSCs with ESCs at the PAS level in comparison with fibroblast and EB cell lines, which formed separate clusters in a PAS heat map. Furthermore, we built a new PAS-based quality score that was able to identify impaired iPSC lines. Several iPSC lines that we predicted as having aberrant pathway signaling also showed a poor *in vitro* neural differentiation pattern. However, it is worth pointing out that one iPSC line displaying a lower than average PAS quality score, had an increased differentiation capacity, indicating that above as well as below average differentiation capacity may be detected by PAS quality score evaluation. Thus, these results clearly demonstrated that our algorithm shows promise for assessing iPSC differentiation capacity *in silico*.

LASSO model coefficients identified nine signaling pathways as strong predictors of iPSC versus fibroblast characteristics. Interestingly, whole genome analysis of gene expression during dopaminergic differentiation of human ESC and iPSC lines revealed that among the distinct pathways which are activated during neuronal differentiation, the cAMP signaling pathway was found to play a significant role in the differentiation of dopaminergic neurons. The role of cAMP signaling was confirmed by small molecule perturbation experiments [27], indicating that cAMP and associated signaling molecules promote dopaminergic differentiation of human ESC and iPSC lines. These findings are in line with our LASSO experiments showing that different components of the cAMP signaling pathway, implicated in cell survival and degradation of cell cycle regulators, were strong predictors of iPSC *vs*. fibroblast characteristics, and that the cAMP pathway was also among the top 20 least variable pathways in ESC and iPSC lines, thus, defining a key role of this pathway in stem cell differentiation. One of the major advantages of the signaling pathway analysis bioinformatics algorithms (Regeneration Intelligence, OncoFinder), are their unique ability to quantify perturbations for signaling pathways relative to control samples on a mathematical basis [18, 24]. It was previously shown that when comparing different gene expression datasets generated for the same biological samples using two different experimental techniques, such as next generation sequencing (NGS) and microarray hybridization, only a very weak correlation existed while application of the PAS profiles comparison revealed a high correlation between the NGS and microarray datasets [24] and can significantly reduce errors introduced by transcriptome-wide experimental techniques [24]. Moreover, employing the LASSO algorithm [25, 26] we were able to identify a small number of pathways that can reliably distinguish iPSC lines from fibroblasts. Selecting these pathways will enable to perform quality control of iPSC lines from a very limited amount of transcriptional data, which will speed up future selection and quality control assays significantly. Thus, we propose the novel signal pathway-centric bioinformatics application as a powerful tool to improve quality control of iPSC lines prior to clinical applications.

## MATERIALS AND METHODS

### Gene expression data

The most representative data set available that included 10 or more microarray profiled ESC and iPSC lines and any iPSC differentiation assay linked to the profiled iPSC lines, was chosen from the NCBI GEO and ArrayExpress databases (GSE25970, http://www.ncbi.nlm.nih.gov/geo/query/acc.cgi?acc=GSE25970). Data set GSE25970 contains the information on 43 cell lines profiled using Affymetrix HT Human Genome U133A arrays: 20 human ESC lines, 12 human iPSC lines, six fibroblast cell lines, and five EB cell lines [17]. This data set was utilized to examine PAS scores in ESC and iPSC lines, and fibroblast cell lines served as reference.

### Bioinformatics analysis and transcriptomic expression data pre-processing

All microarray data preprocessing steps were performed in R version 3.1.0 using packages from Bioconductor. Microarray raw data files were background adjusted and normalized with the GCRMA algorithm using the corresponding R packages and quantile normalized. Obtained gene expression values were averaged across all replicates. Heat map generation and hierarchical clustering were performed using R package gplots. LASSO regression was performed with the R package glmnet [26]. Statistical tests and correlation analysis were done with the MS Excel software.

### Pathway Activation Score (PAS) calculation

Preprocessed gene expression data were loaded into Regeneration Intelligence a proprietary software suite developed by Atlas Regeneration, Inc. It enables calculation of the Pathway Activation Score (PAS) for each of the 271 pathways analyzed, *a* value which serves as a quantitative measure of differential pathway activation between the two states. This software suite is a cloud based implementation of topological gene expression aggregation algorithm [18], optimized for the needs of stem cells research and regenerative medicine. Briefly, Regeneration Intelligence is a topology based gene expression aggregation method. The algorithm utilizes the following formula to evaluate pathway activation:

$${\text{PAS}}_{p}=\sum_{n}{\text{ARR}}_{\text{np}}\cdot {\text{BTIF}}_{n}\cdot \text{lg}\left({\text{CNR}}_{n}\right)$$

Here, CNRn is the ratio of the expression level of a gene n in the case sample and in the control; BTIFn is *a* value of beyond tolerance interval flag, which equals 0 or 1; and ARR (activator/repressor role) serves as a discrete value which equals to the following numbers: −1, −0.5, 0, 0.5 or 1 depending on the topological role of the particular gene/protein in the signaling pathway p, respectively. Results for the 271 pathways PAS that were obtained for each sample of ES and iPS cell lines can be found in supplement materials (Supplemental Excel file 1).

### PAS score calculation in iPSC lines

In order to obtain an iPSC line differentiation assessment we established a reference PAS value corridor using ESC line PAS as a gold standard. The ESC PAS quality score was set as average of PAS values across all ESC lines ± SD of the same PAS across all ESC lines for each pathway. For each of the 12 iPSC lines we calculated the number of signaling pathways with PAS values inside of the previously described ESC reference corridor. We used the PAS-based quality score as a metric to identify iPSC outliers that may be functionally impaired.

## SUPPLEMENTARY MATERIAL FIGURE AND TABLE





## References

[1] Takahashi K, Tanabe K, Ohnuki M, Narita M, Ichisaka T, Tomoda K, Yamanaka S (2007). Induction of pluripotent stem cells from adult human fibroblasts by defined factors. Cell.

[2] Thomson J. A, Itskovitz-Eldor J, Shapiro S. S, Waknitz M. A, Swiergiel J. J, Marshall V. S, Jones J. M (1998). Embryonic stem cell lines derived from human blastocysts. Science.

[3] Zhu Z, Huangfu D (2013). Human pluripotent stem cells: an emerging model in developmental biology Development.

[4] Wu S. M, Hochedlinger K (2011). Harnessing the potential of induced pluripotent stem cells for regenerative medicine. Nat. Cell Biol.

[5] Marchetto MC, Carromeu C, Acab A, Yu D, Yeo GW, Mu Y, Chen G, Gage FH, Muotri AR (2010). A model for neural development and treatment of Rett syndrome using human induced pluripotent stem cells. Cell.

[6] Nguyen HN, Byers B, Cord B, Shcheglovitov A, Byrne J, Gujar P, Kee K, Schüle B, Dolmetsch RE, Langston W, Palmer TD, Pera RR (2011). LRRK2 mutant iPSC-derived DA neurons demonstrate increased susceptibility to oxidative stress. Cell Stem Cell.

[7] Kamao H, Mandai M, Okamoto S, Sakai N, Suga A, Sugita S, Kiryu J, Takahashi M (2014). Characterization of human induced pluripotent stem cell-derived retinal pigment epithelium cell sheets aiming for clinical application. Stem Cell Reports.

[8] Seki T, Fukuda K (2015). Methods of induced pluripotent stem cells for clinical application. World J Stem Cells.

[9] Gore A, Li Z, Fung HL, Young JE, Agarwal S, Antosiewicz-Bourget J, Canto I, Giorgetti A, Israel MA, Kiskinis E, Lee JH, Loh YH, Manos PD (2011). Somatic coding mutations in human induced pluripotent stem cells. Nature.

[10] Hussein SM, Batada NN, Vuoristo S, Ching RW, Autio R, Närvä E, Ng S, Sourour M, Hämäläinen R, Olsson C, Lundin K, Mikkola M, Trokovic R (2011). Copy number variation and selection during reprogramming to pluripotency. Nature.

[11] Laurent LC, Ulitsky I, Slavin I, Tran H, Schork A, Morey R, Lynch C, Harness JV, Lee S, Barrero MJ, Ku S, Martynova M, Semechkin R (2011). Dynamic changes in the copy number of pluripotency and cell proliferation genes in human ESCs and iPSCs during reprogramming and time in culture. Cell Stem Cell.

[12] Mayshar Y, Ben-David U, Lavon N, Biancotti JC, Yakir B, Clark AT, Plath K, Lowry WE, Benvenisty N (2010). Identification and classification of chromosomal aberrations in human induced pluripotent stem cells. Cell Stem Cell.

[13] Feng Q, Lu SJ, Klimanskaya I, Gomes I, Kim D, Chung Y, Honig GR, Kim KS, Lanza R (2010). Hemangioblastic derivatives from human induced pluripotent stem cells exhibit limited expansion and early senescence. Stem Cells.

[14] Lister R, Pelizzola M, Kida YS, Hawkins RD, Nery JR, Hon G, Antosiewicz-Bourget J, O'Malley R, Castanon R, Klugman S, Downes M, Yu R, Stewart R, Ren B, Thomson JA, Evans RM, Ecker JR (2011). Hotspots of aberrant epigenomic reprogramming in human induced pluripotent stem cells. Nature.

[15] Hu BY, Weick JP, Yu J, Ma LX, Zhang XQ, Thomson JA, Zhang SC (2010). Neural differentiation of human induced pluripotent stem cells follows developmental principles but with variable potency. Proc Natl Acad Sci U S A.

[16] Mallon BS, Hamilton RS, Kozhich OA, Johnson KR, Fann YC, Rao MS, Robey PG (2014). Comparison of the molecular profiles of human embryonic and induced pluripotent stem cells of isogenic origin. Stem Cell Res.

[17] Bock C, Kiskinis E, Verstappen G, Gu H, Boulting G, Smith ZD, Ziller M, Croft GF, Amoroso MW, Oakley DH, Gnirke A, Eggan K, Meissner A Reference Maps of human ES and iPSC cell variation enable high-throughput characterization of pluripotent cell lines. Cell.

[18] Buzdin AA, Zhavoronkov AA, Korzinkin MB, Venkova LS, Zenin AA, Smirnov PY, Borisov NM (2014). Oncofinder, a new method for the analysis of intracellular signaling pathway activation using transcriptomic data. Front Genet.

[19] Borisov NM, Terekhanova NV, Aliper AM, Venkova LS, Smirnov PY, Roumiantsev S, Korzinkin MB, Zhavoronkov AA, Buzdin AA (2014). Signaling pathways activation profiles make better markers of cancer than expression of individual genes. Oncotarget.

[20] Lezhnina K, Kovalchuk O, Zhavoronkov AA, Korzinkin MB, Zabolotneva AA, Shegay PV, Sokov DG, Gaifullin NM, Rusakov IG, Aliper AM, Roumiantsev SA, Alekseev BY, Borisov NM, Buzdin AA (2014). Novel robust biomarkers for human bladder cancer based on activation of intracellular signaling pathways. Oncotarget.

[21] Aliper AM, Csoka AB, Buzdin A, Jetka T, Roumiantsev S, Moskalev A, Zhavoronkov A (2015). Signaling pathway activation drift during aging: Hutchinson-Gilford Progeria Syndrome fibroblasts are comparable to normal middle-age and old-age cells. Aging (Albany NY).

[22] Makarev E, Cantor C, Zhavoronkov A, Buzdin A, Aliper A, Csoka AB (2014). Pathway activation profiling reveals new insights into age-related macular degeneration and provides avenues for therapeutic interventions. Aging (Albany NY).

[23] Zhu Q, Izumchenko E, Aliper AM, Makarev E, Paz K, Buzdin A, Zhavoronkov A, Sidransky D (2015). Pathway activation strength is a novel independent prognostic biomarker for cetuximab sensitivity in colorectal cancer patients. Human Genome Variation.

[24] Buzdin AA, Zhavoronkov AA, Korzinkin MB, Roumiantsev SA, Aliper AM, Venkova LS, Smirnov PY, Borisov NM (2014). The OncoFinder algorithm for minimizing the errors introduced by the high-throughput methods of transcriptome analysis. Front Mol Biosci.

[25] Kukreja Sunil L., Lofberg Johan, Brenner Martin J. A least absolute shrinkage and selection operator (LASSO) for nonlinear system identification 2006. https://archive.org/details/nasa_techdoc_20060011038.

[26] Friedman J, Hastie T, Tibshirani R (2010). Regularization Paths for Generalized Linear Models via Coordinate Descent. J Stat Softw.

[27] Momčilović O, Liu Q, Swistowski A, Russo-Tait T, Zhao Y, Rao MS, Zeng X (2014). Genome wide profiling of dopaminergic neurons derived from human embryonic and induced pluripotent stem cells. Stem Cells Dev.

