# Supplementary material for: Quantifying signaling pathway activation to monitor the quality of induced pluripotent stem cells
Source: Oncotarget. 2015 Aug 22;6(27):23204–12. doi: 10.18632/oncotarget.4673 (PMC4695112; doi:10.18632/oncotarget.4673)
Supplement: Supplementary file 1 [file oncotarget-06-23204-s001.pdf]

SUPPLEMENTARY FIGURE AND TABLE

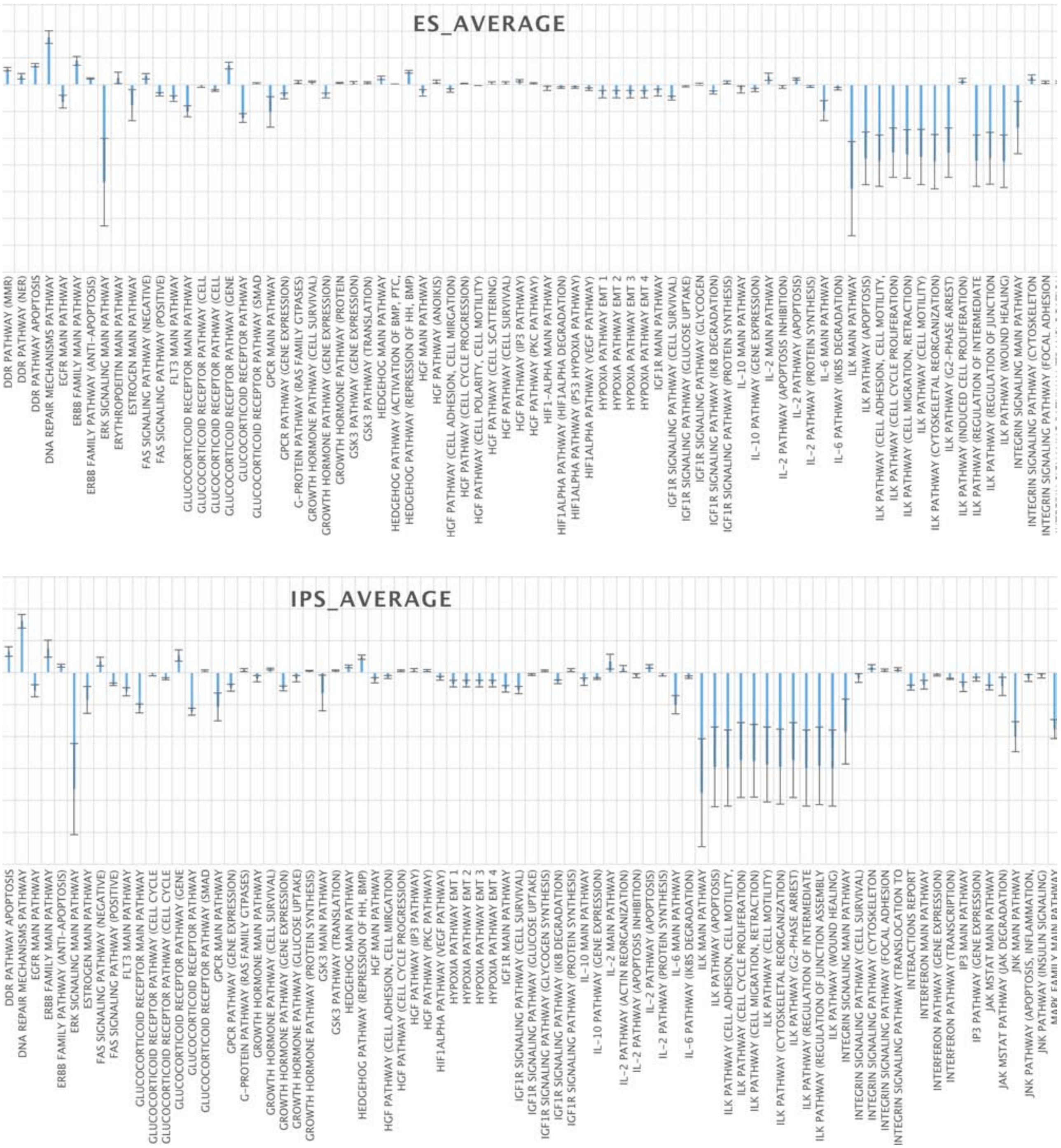

Supplementary Figure S1: Pathway Activation Score (PAS) distribution in ESC and iPSC lines. PAS values were calculated for ES and iPS cell lines from GSE25970. **A.** ESC lines. **B.** IPSC lines. Blue bars show mean PAS values ± SD; only pathways with false discovery rate $q < 0.05$ are shown.

Supplementary Table S1: Raw PAS data for ESC and iPSC lines derived from the GSE25970 dataset. Data for 20 ESC lines are presented in Excel sheet ES tTest PAS1, and data for 12 iPSC lines are shown in Excel sheet iPS tTest PAS1.
